# Experimental immune challenges reduce the quality of male antennae and female pheromone output

**DOI:** 10.1038/s41598-022-07100-y

**Published:** 2022-03-04

**Authors:** Hieu T. Pham, Mark A. Elgar, Emile van Lieshout, Kathryn B. McNamara

**Affiliations:** 1 School of BioSciences, The University of Melbourne, Parkville, VIC 3010 Australia; 2 Department of Entomology, Faculty of Agronomy, Vietnam National University of Agriculture, Hanoi, Vietnam

**Keywords:** Ecology, Evolution

## Abstract

Sexual signalling is a key feature of reproductive investment, yet the effects of immune system activation on investment into chemical signalling, and especially signal receiver traits such as antennae, are poorly understood. We explore how upregulation of juvenile immunity affects male antennal functional morphology and female pheromone attractiveness in the gumleaf skeletonizer moth, *Uraba lugens*. We injected final-instar larvae with a high or low dose of an immune elicitor or a control solution and measured male antennal morphological traits, gonad investment and female pheromone attractiveness. Immune activation affected male and female signalling investment: immune challenged males had a lower density of antennal sensilla, and the pheromone of immune-challenged females was less attractive to males than their unchallenged counterparts. Immune challenge affected female investment into ovary development but not in a linear, dose-dependent manner. While there was no effect of immune challenge on testes size, there was a trade-off between male pre- and post-copulatory investment: male antennal length was negatively correlated with testes size. Our study highlights the costs of elaborate antennae and pheromone production and demonstrates the capacity for honest signalling in species where the costs of pheromone production were presumed to be trivial.

## Introduction

Immune system maintenance and up-regulation is energetically costly^1–3^, so organisms must allocate their finite resources between immune defence and other traits, such as reproduction^4–7^. Theoretical and empirical research reveals phenotypic and evolutionary trade-offs between immune investment and a suite of pre-copulatory and post-copulatory sexual traits [for a review, see^5,8^]. While immune activation has an impact on sexual signalling in both acoustic^9–11^ and visual sensory modalities^12,13^, the impact on chemical signalling is less well understood^14–18^, despite being the dominant and ancestral modality of sexual signalling^19^.

Studies documenting trade-offs between immunity and chemical signalling typically focus on the signaller’s perspective^14–18,20^. Trade-offs between immune investment and chemical-receiving structures (antennae) have not been examined, despite the costs of maintaining these structures^21,22^ and their obvious role in reproduction through mate location and reception of sensory information used in mate assessment. Antennae length and the number of sensilla (chemoreceptors) they bear are important determinants of chemical signalling success^23–25^. In the Lepidoptera, female moths release minute quantities of sex-pheromone^26,27^, so selection may favour males with antennal morphology, including antennal length and sensilla numbers, that optimises odorant-receptor interactions^24,28,29^. For example, male gumleaf skeletonizer moths, *U. lugens,* with long antennae are better able to locate younger females^30^ with a greater residual reproductive value^31^, and male neriid flies *Telostylinus angusticollis* with relatively longer antennae were more successful at acquiring mates^32^. The functional morphology of antennal traits varies facultatively in response to diet or nutrient limitation^33^, and to population demography^34^. Since sensilla are costly and condition dependent^28^, males may trade-off investment in these traits against immune function. Thus, the potential for infection by parasite and/or pathogens may provide a mechanism for maintaining the observed intra-specific variation in antennal investment, as occurs in other sexually selected traits^35^.

Sex pheromones are commonly used by females to reveal their location to males, and the nature of these signals can influence both the number and quality of prospective mating partners^19,24^. Historically, the costs of producing sex pheromones was considered trivial^36–38^, but accumulating evidence indicates that female pheromone quality and calling (pheromone-releasing behaviour) are condition dependent^20,39,40^ and exhibit significant plasticity^41,42^. Nevertheless, Barthel et al.^16^ provide rare evidence of a trade-off between female sex pheromone and immunity: the composition of sex pheromone produced by female tobacco budworm moths, *Heliothis virescens*, changed following injection with a pathogenic immune elicitor, with females experiencing reduced mating success.

We examine the trade-offs between immune investment and female chemical signalling and male sensory structures in the gumleaf skeletonizer moth, *Uraba lugens,* (Lepidoptera: Nolidae), a capital breeding species. The poor flight capacity of females^43^ means their mating success depends largely on the attractiveness of their sex pheromone. Male reproductive success is primarily determined by their longevity and speed of locating receptive females because female re-mating rates are low (18.34%), and males mate once per night only (personal observation). Female pheromone investment is facultatively adjusted in response to population density^42^ and age^44^, and male *U. lugens* adjust their investment in pre-copulatory (antennae) and post-copulatory (testes size) sexually selected traits in response to population demography. For example, males reared at low population densities produce larger antennae, thereby improving their mate detection^30,34^. Specifically, we address two questions: first, is there a dose-dependent impact of immune challenges on trade-offs between traits associated with pre- and post-copulatory sexual selection; and second, what is the impact of immune trade-offs on life-history traits, including body size and longevity?

## Materials and methods

### Insect culturing

Australian gumleaf skeletonizer moth, *U. lugens,* were collected as eggs from multiple egg batches and locations in Melbourne, Australia. Larvae were maintained under constant conditions (15L:9D light:dark; 22.5 °C; 70% humidity) in plastic containers (40–50 individuals per 1 L container) until the fifth instar, after which individuals were moved to a 1 L container (10 individuals/container). Containers were supplied with fresh, mature leaves of *Eucalyptus* spp.

### Immune assays

On the day of their final-instar moult (males: 9th instar; females: 10th instar), individuals were haphazardly collected from the stock population, weighed (to the nearest 0.1 mg) and allocated to one of three immune-challenge treatments: a ‘high’ dose of a non-pathogenic immune elicitor, a lipopolysaccharide (LPS) derived from *Serratia marcescens* (Sigma-Aldrich L6136); a ‘low’ dose of LPS; or a solution of an isotonic insect ringer (Sigma- Aldrich G8142) as a control. LPS allows quantification of the costs of immune responses, without the confounding effects of physiological sickness. LPS induces upregulation of a number of invertebrate immune effector systems, consequently reducing investment in a range of reproductive traits^45,46^. LPS doses were modified from McNamara et al.^45^, scaled appropriately for species-and sex-specific body mass. We used sex‐specific mean weights of a cohort of final‐instar *U. lugens* to calculate the relative dose for this species (high dose = 0.08 μg of LPS/mg of larvae; low dose = 0.06 μg of LPS/mg). Accordingly, males were injected with either 3.80 µg or 2.66 µg and females with 5.99 µg or 4.19 µg of LPS, for high and low doses, respectively. LPS was dissolved in 1.5 µl of ringer and injected in the rear proleg using a Hamilton micro-syringe (7632-01) with a 33-gauge needle (7803-05). Control treatment individuals were injected with 1.5 µl of ringer. Larvae were maintained individually in 1L plastic containers and fed with *Eucalyptus* spp. leaves until pupation. Pupae were transferred to individual vials (40 × 60 mm, 120 ml) under standard laboratory conditions until adult eclosion.

### Immune challenge, juvenile development, longevity and adult body size

In total, 263 females (control = 84, low-dose = 67, high-dose = 112) and 322 males (control = 95, low-dose = 132, high-dose = 95) were assessed daily for survival. Of those that survived, 92 females (control = 39, low-dose = 25, high-dose = 28) and 115 males (control = 34, low-dose = 49, high-dose = 32) were assessed for adult body size (wing size) and longevity (from adult eclosion until death). Wing length was measured as an index of body size [following^42^].

### Immune challenge and male pre- and post-copulatory reproductive investment

In a separate experiment, we assessed the effect of immune challenge on male pre- and post-copulatory reproductive investment, at two days following adult eclosion. Each male’s left antenna was removed and mounted on a 12.6 mm scanning electron microscopy (SEM) stub (Proscitech G040) and imaged on a FEI Quanta 200F SEM (spot size = 2.0; pressure = 0.80 mbar; voltage = 10.0 kV) (Bio21 Institute, The University of Melbourne, Australia). We measured antennal length (from tip to scape), flagellomere (segment) number and the density of trichodea sensilla, given their role in olfactory mate choice in the Lepidoptera^47^ (see Supplementary materials for details) using ImageJ^48^. The average sensilla density for each male, based on the sensilla density of the 1st (from the tip) and 35th flagellomeres, was calculated by dividing the total number of trichodea sensilla by the total area in μm^2^ of those flagellomeres.

Testes were removed and placed on a glass slide and photographed using a Sony camera (ILCE-QX1) mounted on an Olympus microscope (SZX16) at × 5 magnification. The area of the testes was measured using the trace function (and standardised using a calibration slide) using ImageJ^48^. All male and female morphological measurements were obtained blind to the experimental treatment.

### Immune challenge and female reproductive investment

In a separate experiment, we assessed the effect of immune challenge on female mate attraction investment and fecundity, by measuring female pheromonal attractiveness and ovary mass.

The effect of immune challenge on female pheromone quality was evaluated using a Perspex y-maze olfactometer (for details, see Fig. S2; Supplementary materials). A standardised, continuous air flow from a single source was introduced into each chamber located at the end of each arm of the Y-maze and on to the receiving male. The chamber containing the female was sealed with a fine mesh, preventing tactile and visual communication with the male; thus, males assessed female attractiveness according to her pheromone only. A single female (LD or HD) was placed in one container and a single control female was placed in the other container and left to acclimate for 1 h. Both females were ≤ 48 h post eclosion. Once both females commenced calling, a virgin, stock (non-experimental) male (≤ 36 h post eclosion) was introduced into the central arm of the Y-maze, and was deemed to have responded when he either walked or flew toward the airflow, travelling at least 5 cm into one of the arms of the y-maze and remaining there for more than 1 min. Males that did not initiate movement within 30 min were replaced with another male. Males were given 60 min to make a choice or were excluded. Males were used once only. Trials were conducted during the middle of the scotophase, with an overhead red light used for illumination. The location of the treatment and control females was alternated between trials to minimise potential positional bias.

Immediately following completion of the trial, females were frozen at − 20 °C. Ovary mass was used as an index of fecundity. Ovaries were dissected out, all extraneous tissue carefully removed, and weighed using a microbalance (Mettler Toledo XS205). Wing length was also measured (as above).

### Statistical analysis

Statistical analyses were conducted, and all figures created, in RStudio (v. 1.2.5042)^49^. The effect of immune-challenge treatment on survival was explored using a Generalized Linear Model with a binomial error distribution. The effect of immune challenge treatment on adult longevity and wing size were explored using General Linear Models.

Male testes size and antennal morphology were examined using Principal Components Regression. Antennal length, antennal segment number, sensilla density, and testes size were incorporated into a Principal Components Analysis. The PCs were then used as dependent variables in General Linear Models, with male body size as a covariate.

Male olfactory preference trials were analysed in R using a Generalized Linear Mixed Model with a binomial error distribution (GLMM—package ‘lme4’) (Bates et al. 2015). All treatment females were used twice, each time with a different control female. Thus, female identity was included as a random effect. We examined the likelihood that males would choose the female in the left chamber of the Y-maze, with the treatment of the left-chamber female and her relative weight as predictor variables.

Ovary mass is strongly predicted by adult body mass (F_1,73_ = 34.58, *p* < 0.0001). Thus, we used residual ovary mass as the response variable in a General Linear Model to explore the impact of immune treatment on female gonad investment.

The significance of any differences between treatment means after ANOVA modelling was established by determining whether the 95% confidence intervals overlapped with zero. Model coefficients and their standard errors are provided for continuous variables to show the directional relationships.

## Results

### The effect of immune challenge on survival, longevity and adult wing size

**Figure 1:**
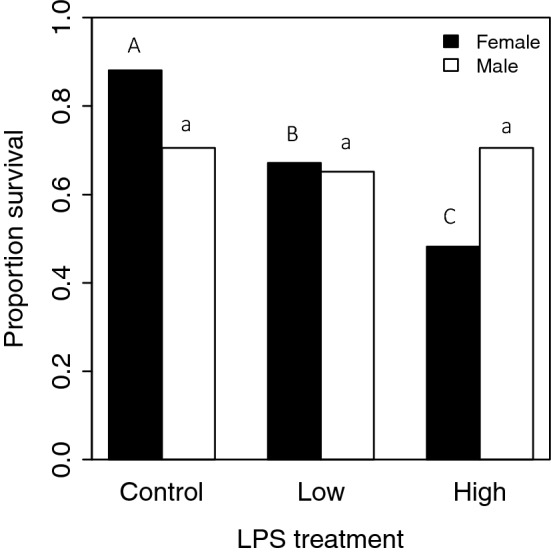
Proportion of males and females from each immune challenge treatment to survive until adult eclosion. Males and females were analysed separately. Different letters (females = uppercase, males = lowercase) denote differences between treatments.

We examined the effect of LPS dose and larval body mass on the likelihood of survival until adult eclosion. Low LPS treatment (LD) females had lower survival to eclosion compared with control females, as did high LPS treatment (HD) females compared with LD females (Table 1; Fig. 1). Male survival was not affected by the immune challenge treatment, but increased significantly with larval mass (Table 1, Fig. 1).

**Table 1:** Models examining the impact of immune challenge treatment on survival, wing size, and adult longevity. For analyses of the likelihood of survival until adult eclosion and wing size, larval body mass was used as a covariate. For analysis of adult longevity, wing size was used as a covariate.

Sex	Factor	Model					
		Survival		Wing size		Longevity	
Males	Treatment	χ^2^_2_ = 3.93	*p* = 0.14	χ^2^_2_ = 0.39	*p* = 0.82	χ^2^_2_ = 9.93	***p* = 0.01**
	Body size	χ^2^_1_ = 23.94	***p* < 0.0001**	χ^2^_1_ = 5.48	***p* = 0.02**	χ^2^_1_ = 3.89	***p* = 0.048**
Females	Treatment	χ^2^_2_ = 37.18	***p* < 0.0001**	χ^2^_2_ = 7.68	***p* = 0.02**	χ^2^_2_ = 10.32	***p* = 0.006**
	Body size	χ^2^_1_ = 0.56	*p* = 0.45	χ^2^_1_ = 3.18	*p* = 0.07	χ^2^_1_ = 0.27	*p* = 0.60

**Figure 2:**
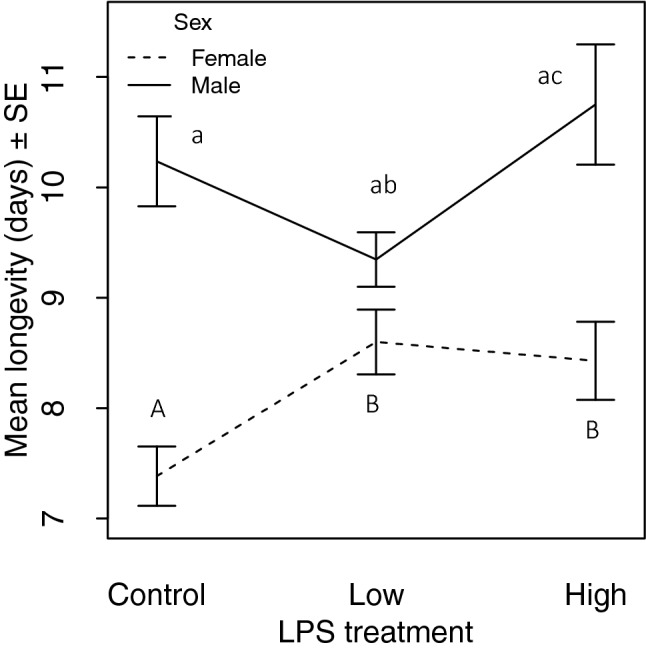
Mean adult longevity ± SE of males and females that received an immune challenge (Low or High dose) or a Control treatment. Males and females were analysed separately. Different letters (females = uppercase, males = lowercase) denote differences between treatments.

For analyses of adult longevity, we examined the effect of LPS dose and wing size separately for males and females. Adult female longevity was greater for immune challenged than control females (Table 1; Fig. 2) but was not affected by adult wing size (Table 1). Adult male longevity increased with adult wing size (Table 1) and was greater for HD compared with LD males (Table 1; Fig. 2).

For adult wing size, we explored the effect of LPS dose and the mass of the larva at the time of injection (larval mass) separately for males and females. Adult wing size was measured for 92 females (control = 39; LD = 28, HD = 25). LD females were smaller than control females, but no other differences were detected (Table 1). Body size was not affected by larval mass (mean ± standard error adult wing length (mm); control = 10.60 ± 0.09; LD = 10.25 ± 0.09; HD = 10.41 ± 0.08; Table 1). Wing size was measured for 92 males (control = 34; LD = 49, HD = 32). Male wing size was not affected by the immune challenge treatment but increased with larval mass (mean ± standard error adult wing length (mm); control = 7.81 ± 0.06; LD = 7.81 ± 0.06; HD = 7.71 ± 0.07; Table 1).

### Immune challenge and male pre- and post-copulatory reproductive investment

PCA of male antennal traits and testes size returned two axes of variation (PCs) with eigenvalues > 1.0, which collectively explained 67% of the variation in the recorded traits (Table 2). PC1 was positively weighted by variables describing the length of the antenna (total length and number of flagellomeres) and negatively by male testes size, indicating a trade-off between these pre- and post-copulatory traits (partial correlation between testes size and antennae length (controlling for wing size): r = − 0.32 n = 76, *p* = 0.005). PC2 was weighted positively by the density of sensilla (Table 2).

**Table 2:** Summary of fit and loadings of PCA and mean ± standard errors (SE) for male antennal morphology and testes size for males from different immune challenge treatments.

	Mean ± SE				
	Control	Low dose	High dose	PC1	PC2
Eigenvalue				1.63	1.05
% variance explained				40.85	26.26
n	26	27	23		
Antennal length (mm)	5.27 ± 0.04	5.23 ± 0.04	5.33 ± 0.05	0.60	−0.23
Antennae segments	49.61 ± 0.50	49.11 ± 0.29	49.57 ± 0.54	0.62	0.27
Sensilla density (μm^−2^)	5.29 ± 0.09 × 10^–3^	5.01 ± 0.12 × 10^–3^	5.01 ± 0.09 × 10^–3^	−0.20	0.87
Testes size (mm^2^)	0.26 ± 0.01	0.27 ± 0.01	0.27 ± 0.01	−0.46	−0.33

**Figure 3:**
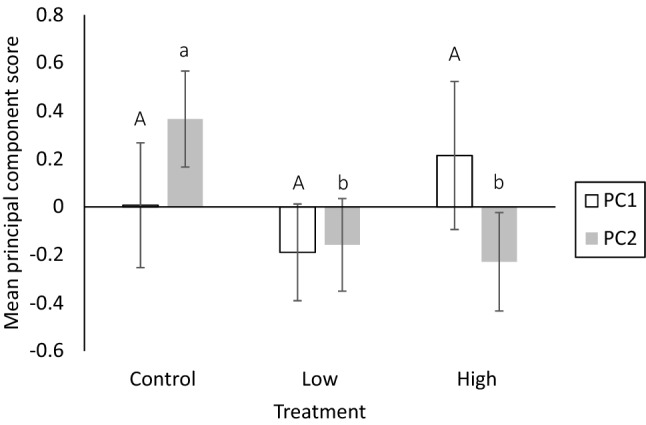
Mean principal component scores ± SE for males that received an immune challenge (Low or High dose) or a Control treatment. PC1 and PC2 were analysed separately. Different letters (PC1 = uppercase, PC2 = lowercase) denote differences between treatments.

PC1, which describes male antennal length and testes size, was not affected by the dose of immune elicitor (F_2,74_ = 0.56, *p* = 0.57; Fig. 3), but increased with male wing size (F_1,74_ = 4.42, *p* = 0.04).

PC2, which describes sensilla density, significantly differed between HD and LD males compared with control males (F_2,74_ = 3.18, *p* = 0.047; Fig. 3). The principal component loadings reveal that this was due to males from the immune challenge treatments having lower sensilla density. Furthermore, PC2 decreased with male wing size (F_1,74_ = 14.35, *p* = 0.0003).

### Immune challenge and female reproductive investment

Eighteen males made a successful choice between HD and control females, with males preferring the pheromone of control females (control = 12; HD = 6; χ^2^_1_ = 6.08, *p* = 0.01), and for heavier females (χ^2^_1_ = 7.34, β = 24.88 (9.32), *p* = 0.01). Eighteen males made a successful choice between LD and control females, but showed no preference according to treatment (control = 11; low dose = 7; χ^2^_1_ = 1.16, *p* = 0.28) or body mass (χ^2^_1_ = 0.78, β = 1.12 (1.26), *p* = 0.37).

**Figure 4:**
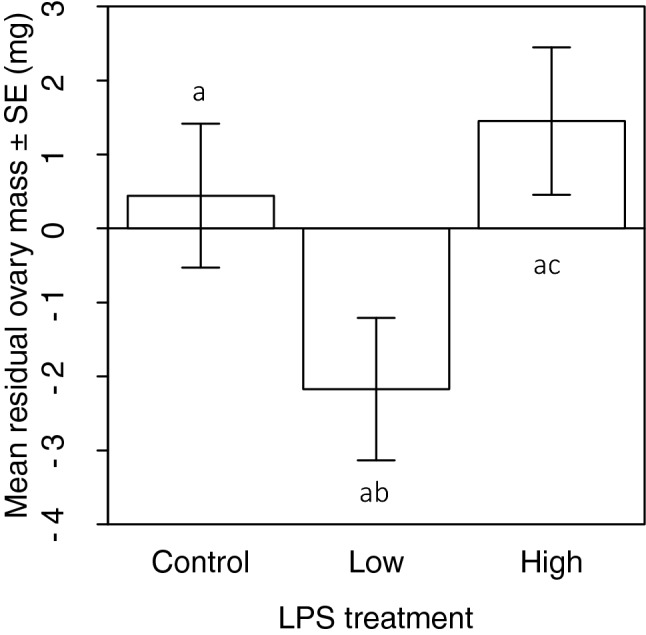
Mean ± standard error residual ovary mass of females received an immune challenge (Low or High dose) or a Control treatment. Different letters denote differences between treatments.

Seventy-five females were assessed for ovary mass (low dose = 22, high dose = 24; control = 29). Female ovary mass increased with female mass (F_1,73_ = 34.58, β = 1.81 (0.31), *p* < 0.0001). Thus, female residual ovary mass (regressed on adult body mass) was used to explore the impact of immune challenge on female gonadic investment. Residual ovary mass differed significantly only between HD and LD females (F_2,72_ = 3.30, *p* = 0.04; Fig. 4).

## Discussion

Our experiments demonstrate that immune investment impacts pre- and post-copulatory reproductive investment and life-history traits in both male and female gumleaf skeletonizer moths, but not in a consistently dose-dependent manner. In particular, our novel results demonstrate that chemical communication is costly for both males and females: immune trade-offs affect male sensory structures, highlighting the costs of elaborate antennae, and the attractiveness of the female pheromone (following a high-dose immune elicitor) declines following immune challenge. Females adjust pheromone production in response to physiological stressors, consistent with the view that pheromone production is costly and therefore a reliable signal. These trade-offs in both male and female sexual signalling are balanced by additional changes in life-history and reproductive traits.

Juvenile immune challenge in *U. lugens* has a significant impact on male mate searching, affecting investment into functional antennal morphology and the time available to find receptive females (male adult lifespan). Current evidence for the costs associated with mate detection through olfactory sensory modalities is largely non-experimental or indirect: a positive correlation between male antennal and body size has been documented across species of lepidoptera^28^, and more generally studies indicate that antennal sensilla require significant and costly neural innervation^21,50^. The lower density of antennal sensilla of immune-challenged males, not accompanied by compensatory changes in antennal length, suggests that the capacity for pheromone detection is weaker in these males^51^, thereby compromising their mate searching success^28,52^. Our results demonstrate that male chemoreceptor density is costly, consistent with the view that releasing minute quantities of pheromone is a female strategy to attract high-quality males^28,53^. Furthermore, we show that increased investment into immunity, generated by parasitic or pathogenic infection, can provide a mechanism for variation in male sensory structures, which are under strong selection. Indeed, such trade-offs may persist into the next generation, as has been demonstrated for other reproductive traits^54^.

Honest signals are assumed to be costly, and the sex pheromone of *U. lugens* is likely to function as an honest signal of female quality: our results demonstrate a cost to pheromone production that are consistent with previous studies demonstrating that females adjust their pheromone investment according to the socio-sexual environment^42^. The historical assumption that female pheromone production, particularly in lepidopterans, is not costly because only minute chemical titres are released is recently challenged by evidence of condition dependent pheromone production^39,41^. Our study provides more compelling empirical support for this view by demonstrating clear trade-offs between immunity and female sexual signalling (see also^16^) that translates into attractiveness to males. Pheromone production in the Lepidoptera is low because of constraints on biosynthesis, storage and gland structure^55^. We were unable to replicate the published methodology for chemical quantification of the contents of female pheromone glands in *U. lugens*^56^, but the observed patterns of male attraction may derive from qualitative and/or quantitative differences in the pheromone profile.

Immune challenges appear to have a more consistent negative effect on post-copulatory reproductive investment in males than females in diverse species: there is a reduction in the size^57^, viability^46^, and number^45^ of male spermatophores, but the response by females is less clear, with lower^58,59^, similar^45^, or higher reproductive output^60^ recorded. Immune-challenged male *U. lugens* did not appear to reduce their investment into testes size, which may be unsurprising in this monandrous [sensu^61^] species: sperm competitive strategies are likely to be less important, given their low mating frequency^44^ arising from limited mating opportunities. On the other hand, inferences about immunity trade-offs with female egg production in *U. lugens* are less clear: females that received a high dose of LPS had a greater ovary mass than LD females, but not control females. This is surprising because egg production is a highly resource-dependent trait, and trade-offs arising from immune challenges may be especially pronounced in capital breeders, such as *U. lugens*, where individuals acquire all their resources for reproduction in the juvenile stage. Perhaps immunity-fecundity trade-offs are shaped by both mating system and the type of immune elicitor used.

Conventional theory predicts trade-offs between precopulatory traits (ornaments and armaments) and between post-copulatory traits (testes and ejaculates)^62^, although empirical investigations have focused primarily on male ornaments and armaments^63^. Early sperm competition models noted trade-offs between ejaculates and mate searching investment^64^, and while negative relationships between testes size and mate searching ability have been reported^65,66^, the potential for trade-offs between testes and organs of sense has rarely been explored, despite their primary function in mate searching and mating success. One study failed to find a trade-off between antennal investment and testes size in the monandrous painted apple moth, *Teia anartoides*^67^, but the present experiments revealed a clear trade-off between male mate searching investment (antennal length) and testes size in the similarly monandrous *U. lugens*, regardless of experimental treatment.

The high reproductive costs paid by males in this mating system, evidenced by their lengthy matings (> 1.5 h; personal observation) and low mating frequency^44^, may affect the trajectory of development in immune-challenged individuals. Like most Lepidoptera, *U. lugens* mate once per day only, so mating frequency is tightly linked to longevity. The reduced investment into mate-detecting sensory structures by immune-challenged males may be balanced by increased longevity, as HD males had greater longevity than LD males, albeit not different from control males. Female *U. lugens* also increased longevity following immune challenge, but this was balanced by a greater mortality at the pre-adult stage (for both doses of immune elicitor) and lower reproductive output (for HD females). That female, but not male, mortality increased after receiving LPS suggests a sexual dimorphism in immunity in this species, with females having a lower immunity. While this pattern contradicts that predicted by traditional Bateman gradients of immunity^68^, it may reflect the significant male reproductive investment in this moth species and others with comparable life-histories^16^. Clearly, a greater understanding of the degree of dimorphism in constitutive and expressed immunity is needed, and especially in how it affects those traits associated with mate discovery.

Our results also demonstrate inconsistent behavioural and morphological responses to the dose of immune elicitor used. Hormesis is a phenomenon where individuals respond more strongly to low, compared with high doses, of a biological stressor^69,70^, including LPS. While hormesis may explain why LD, but not HD, females had lower survival and smaller body sizes than Control females, it offers a less satisfactory explanation for why some traits (male longevity and ovary mass) differed between treatments, but not with the Controls. Perhaps a broader range of LPS doses, and quantification of male and female immune response to the different doses may clarify these inconsistent trait responses.

In conclusion, we reveal that the functional morphology of male antennae and, therefore, their capacity to locate mates is altered by an immune challenge. We also demonstrate that immune stressors reduce female pheromone quality and attractiveness. thereby highlighting the costs and condition dependence of male and female sexual signalling and thus a mechanism for variation in reproductive traits that are likely to be under strong selection.

## Supplementary Information

Supplementary Information.
