# Supplementary material for: Experimental immune challenges reduce the quality of male antennae and female pheromone output
Source: Sci Rep. 2022 Mar 4;12:3578. doi: 10.1038/s41598-022-07100-y (PMC8897396; doi:10.1038/s41598-022-07100-y)
Supplement: Supplementary file 1 — Supplementary Information. [file 41598_2022_7100_MOESM1_ESM.docx]

SUPPLEMENTARY MATERIALS

The costs of chemical signalling and reception: immune activation reduces the quality of male antennae and female pheromone output.

We detected six types of male antennal sensilla [following 1], based on their external morphology; trichodea, basiconica, coeloconica, auriculate, chaetica, and styloconica sensilla (Figure S1). Trichodea sensilla were the dominant type of sensilla in all flagellomeres of the male antenna and were the only type found in the antennal branches. Trichodea sensilla have numerous pores, are regarded as olfactory, and have been implicated in mate search in tortricid moths [2]. In contrast, basiconica, coeloconica and auriculate sensilla are involved in host plant odour perception [2, 3], and chaetica and styloconica sensilla do not have pores on the surface and are therefore unlikely to function as olfactory receptors.

Figure S1. Different types of sensilla located on ventral view on one segment: the long sensilla trichodea (Tr); sensilla chaetica (Ch) are similar to Tr but the basal socket is different; sensilla auricillica (Au) and sensilla Basiconica (Bs) have a flat shape and are wide at the base; sensilla coeloconia (Co) are a grooved peg surrounded by 12-16 spines; and sensilla styloconica (St) are peg-like, set on top of a conical cuticular style found at the distal margin of each flagellomere.


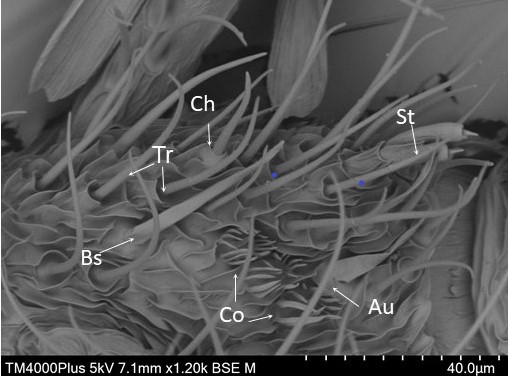


Accordingly, we measured the density of trichodea sensilla only and, for convenience, refer to this as sensilla density. The average sensilla density for each male, based on the density of trichodea sensilla of two flagellomeres (1^st^ and 35^th^ from the antennal tip), was calculated by dividing the total number of trichodea sensilla observed in those two flagellomeres by the total area in μm^2^ of those flagellomeres.

References

1. Hansson B.S. 1995 Olfaction in Lepidoptera. *Experientia* **51**(11), 1003-1027. (doi:10.1007/BF01946910).

2. Triseleva T.A., Safonkin A.F. 2006 Variation in antennal sensory system in different phenotypes of large fruit-tree tortrix Archips podana scop. (Lepidoptera: Tortricidae). *Biol Bull* **33**(6), 568-572. (doi:10.1134/S1062359006060069).

3. den Otter C.J., Schuil H.A., Sander-van Oosten A. 1978 Reception of host-plant adours and female sex pheromone in *Adoxophyes orana* (Lepidoptera: Tortricidae): electrophysiology and morphology *Entomol Exp Appl* **24**(3), 570-578. (doi:https://doi.org/10.1111/j.1570-7458.1978.tb02818.x).

Figure S2. Schematic of the Y-maze apparatus used to assess male preferences for sex pheromones from females that received an immune challenge (High (H) or Low (L) dose) or a Control treatment.

| 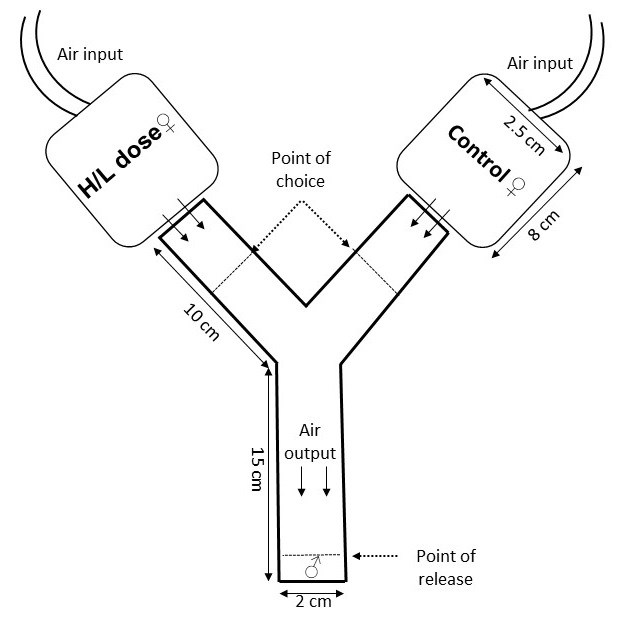 |
| --- |
|  |
